# Supplementary material for: M2 Monocyte Polarization in Dialyzed Patients Is Associated with Increased Levels of M-CSF and Myeloperoxidase-Associated Oxidative Stress: Preliminary Results
Source: Biomedicines. 2021 Jan 16;9(1):84. doi: 10.3390/biomedicines9010084 (PMC7830480; doi:10.3390/biomedicines9010084)
Supplement: Supplementary file 1 [file biomedicines-09-00084-s001.pdf]

**Supplementary data**

| Patient Number | Gender | Age (y) | HD Time/week | Dialyzer | Access | Smoking Status | Diabetes |
|---|---|---|---|---|---|---|---|
| P1 | M | 83 | 12h | HF22 | Hemocath | No | Yes |
| P2 | M | 64 | 10h30 | HF22 | Hemocath | Yes | Yes |
| P3 | M | 64 | 10h30 | FX80 | Hemocath | Yes | No |
| P4 | M | 29 | 12h | HF22 | Hemocath | No | No |
| P5 | M | 88 | 9h | EVO | Hemocath | No | No |
| P6 | F | 88 | 9h | FX80 | FAV | No | No |
| P7 | F | 68 | 9h | HF17 | Hemocath | No | No |
| P8 | F | 88 | 10h30 | Nephral 500 | Hemocath | No | Yes |
| P9 | M | 61 | 12h | FX80 | Hemocath | Yes | Yes |
| P10 | M | 62 | 12h | HF22 | FAV | No | No |
| P11 | M | 59 | 12h | Nephral 500 | Hemocath | Yes | Yes |
| P12 | F | 81 | 12h | HF17 | Hemocath | No | No |
| P13 | F | 94 | 9h | HF17 | Hemocath | No | Yes |
| P14 | F | 87 | 6h | HF22 | Hemocath | No | No |
| P15 | M | 50 | 12h | HF22 | Hemocath | No | No |
| P16 | M | 74 | 10h30 | HF22 | FAV | No | No |
| P17 | M | 59 | 16h | HF22 | Hemocath | Yes | Yes |
| P18 | F | 77 | 9h | HF22 | Hemocath | No | Yes |
| P19 | M | 71 | 9h | EVO | Hemocath | No | No |
| P20 | F | 70 | 12h | HF22 | Hemocath | No | Yes |
| P21 | M | 79 | 10h30 | HF22 | FAV | No | No |
| P22 | M | 63 | 10h30 | HF22 | FAV | No | No |
| P23 | F | 88 | 12h | Nephral 500 | Hemocath | No | Yes |
| P24 | M | 69 | 12h | HF22 | Hemocath | No | Yes |
| P25 | M | 80 | 10h30 | HF17 | Hemocath | No | No |
| P26 | M | 72 | 12h | Nephral 500 | Hemocath | No | Yes |
| P27 | F | 71 | 12h | FX80 | Hemocath | No | No |

**Table S1. Summary of patient data.**

HF22 (polyphenylene): high flux; FX80 (polysulfone): high flux; Nephral 500 (AN69ST): high flux; Hemocath: long-term tunneled catheter; FAV: arterio-venous graft.
